# Assessing Visual Outcomes: A Comparative Study of US-FDA Premarket Approval Data for Multifocal and EDOF Lens Implants in Cataract Surgery

**DOI:** 10.3390/jcm12134365

**Published:** 2023-06-28

**Authors:** Majid Moshirfar, Isabella M. Stoakes, Joshua S. Theis, Kaiden B. Porter, Jordan M. Santos, Tanisha Martheswaran, Carter J. Payne, Phillip C. Hoopes

**Affiliations:** 1 Hoopes Vision Research Center, Hoopes Vision, Draper, UT 84020, USAcjp127@case.edu (C.J.P.); pch@hoopesvision.com (P.C.H.); 2 John A. Moran Eye Center, University of Utah School of Medicine, Salt Lake City, UT 84132, USA; 3 Utah Lions Eye Bank, Murray, UT 84107, USA; 4 Pacific Northwest University of Health Sciences, College of Osteopathic Medicine, Yakima, WA 98901, USA; 5 University of Arizona School of Medicine, Phoenix, AZ 85004, USA; 6 The Johns Hopkins School of Medicine, Baltimore, MD 21205, USA; tmarthe1@jh.edu; 7 Case Western Reserve School of Medicine, Cleveland, OH 44106, USA

**Keywords:** multifocal IOL, extended depth-of-focus (EDOF), US-FDA, PanOptix, Synergy, Symfony, Tecnis, Acrysof, cataract surgery, glasses independence

## Abstract

This study compares the efficacy, safety, and patient-reported outcomes of three intraocular implants (IOL): Tecnis Synergy IOL, AcrySof IQ PanOptix Trifocal, and Tecnis Symfony EDOF IOL. Participants achieving 20/20 or better uncorrected binocular visual acuity were as follows: Synergy—67% distance, 64% intermediate, and 47% near; PanOptix—73% distance, 73% intermediate, and 50% near; and Symfony—63% distance, 75% intermediate, and 22% near. Symfony demonstrated superior intermediate visual acuity compared to Synergy (*p* = 0.0182) for those achieving 20/25 or better. Both Synergy and PanOptix showed superiority over Symfony for near visual acuity (*p* < 0.0001). Halos were statistically more common in Synergy participants compared to PanOptix (*p* = 0.0013) and Symfony (*p* < 0.0001). Each trial lens outperformed its monofocal IOL in terms of independence from glasses or contacts, with Synergy and PanOptix showing statistical significance over Symfony. Comparing contrast sensitivities and defocus curves was challenging due to data variance and as such, standardization of United States Food and Drug Administration (US-FDA) data reporting is key for better comparison of outcomes among different IOL platforms.

## 1. Introduction

Cataracts are a leading cause of vision loss in the United States currently affecting an estimated 17% of Americans. Furthermore, projections indicate that the number of individuals affected by cataracts will exceed 140 million by the year 2050 [1]. However, thanks to proactive screening measures and advancements in surgical and post-surgical interventions, cataracts have become highly treatable and cataract surgery is one of the most common surgeries performed in the United States [2].

Traditionally, cataract surgery involved replacing the cataractous lens with a monofocal intraocular lens (IOL), which provided a single fixed focal point [3]. Due to the capability of these IOLs to be curated to each patient’s ocular anatomy, cataract surgery has evolved into a refractive procedure, akin to laser-assisted in situ keratomileusis (LASIK). Patients increasingly seek optimal visual outcomes post-surgery in addition to the replacement of their lens. However, this approach limits patients’ ability to focus on various distances due to the lack of accommodative ability with a fixed silicone or acrylic IOL. In recent years, studies utilizing newer multifocal and extended depth of field (EDOF) IOL implants have shown promise in restoring near and intermediate acuities while maintaining satisfactory distance acuity [4,5,6,7,8,9,10,11,12].

Multifocal IOLs utilize various optical principles to enhance vision at multiple distances. One type of multifocal IOL design is the diffractive bifocal lens, which incorporates distinct zones on the lens surface that diffract incoming light to form two distinct focal points enhancing both near and distance acuity [13]. Trifocal IOLs further extend this concept by incorporating an additional focal point for intermediate vision, providing enhanced visual acuity at near, intermediate, and distance ranges [14].

EDOF IOLs, on the other hand, utilize a different mechanism to achieve a continuous range of vision. These lenses employ a combination of diffractive and refractive optical properties. By manipulating the wavefront of incoming light, EDOF IOLs extend the depth of focus, allowing patients to achieve satisfactory vision at a range of distances without distinct focal points [15,16]. Tecnis Synergy specifically, uses a proprietary echelette design and achromatic technology via a unique diffractive pattern [17]. This results in a gradual transition from near to distance, reducing the occurrence of visual disturbances associated with traditional multifocal IOLs [18]. However, retinal, optic nerve, and zonular health in addition to pupil size are important factors when a patient is considering a multifocal IOL [19].

In this article, we focus on evaluating the safety and efficacy of three FDA-approved multifocal and EDOF IOLs through their respective Premarket Approvals (PMAs): Acrysof IQ PanOptix Trifocal by Alcon (PMA #P040020/S087), Tecnis Synergy IOL (PMA #P980040/S124), and Tecnis Symfony EDOF IOL (PMA #P980040/S065), both by Johnson & Johnson [20,21,22]. By examining their specific design features, optical properties, and clinical performance, we aim to provide valuable insights to assist surgeons in creating more transparency for their patients when selecting the most suitable intraocular lens.

## 2. Materials and Methods

### 2.1. Data Analysis

The data in this analysis were obtained from three US-FDA PMAs submitted by Acrysof for IQ PanOptix Trifocal and by Tecnis for their Symfony IOL and Synergy EDOF IOL. Due to the significant variation in measurement methods across studies, a direct comparison between lenses for all variables was not feasible. Therefore, this article primarily compares the efficacy of the lenses by assessing monocular best-corrected distance, intermediate and near visual acuity (CDVA, CIVA, CNVA, respectively), monocular and binocular uncorrected distance, intermediate and near visual acuity (UDVA, UIVA, UNVA, respectively). Additionally, defocus and contrast sensitivity curves provided by the companies, glasses/contacts independence, toricity, and the average spherical equivalent (SEQ) targeted by each company were analyzed when the respective data were provided.

For example, the PMAs did not provide the raw data for the defocus curves, which limited our ability to directly compare the curves between the lenses. However, an approximation of two binocular defocus curves (Symfony and PanOptix) could be compared to an average monofocal control lens. To gather data for glasses/contacts independence, each company collected this data using different questionnaires with varying stratifications across platforms. The stratification strategies included assessing time requirements (wearing corrective lenses some of the time vs. never), focus requirements (requiring correction for distance vs. near), or evaluating the number of days in the past week that subjects used correction.

It is important to note that Symfony OptiBlue, the newest technology introduced by the Symfony lens, was not included in the main analyses as it did not have a stand-alone PMA. All patients included in the analysis underwent bilateral cataract and lens replacement surgeries. In most cases, each eye (first and second) was treated as separate entities in the statistical analysis, as recommended by the respective companies. Data regarding uncorrected visual acuity at near, intermediate, and distance were categorized based on the percentage of patients achieving a Snellen visual acuity of “xx/xx or better”. To compare the outcomes between the two lenses, a Fisher Exact test was employed.

### 2.2. Safety Assessment

Each company assessed the occurrence of common “visual disturbances” associated with their respective lens: glare, halo, and starbursts. Data were gathered at 6 months postoperatively from a questionnaire provided by this study. There were some variations among the groups in how these questionnaires were administered and categorized. Some companies measured occurrence while others stratified occurrence by severity or “bothersomeness” through subjective questionnaires. Consequently, direct comparisons between the lenses were not possible due to the variation in questionnaire designs. Instead, the occurrence rates of each disturbance were reported without considering severity or bothersomeness. To compare the studies, the number of patients who reported experiencing these symptoms at any level of severity was aggregated. For instance, if a study reported that five patients did not experience halos, ten experienced mild halos, four experienced moderate halos, and three experienced severe halos, the total number of patients experiencing halos was concluded as seventeen. Each company compared their safety values to international standards or historical rates to evaluate the safety endpoints of the lenses.

### 2.3. Statistical Analysis

Statistical analyses were performed using Microsoft Excel 2016 and IBM’s SPSS Software Version 27. A significance level of *p* < 0.05 was considered statistically significant. The assumption of a normal distribution was made for the data as the raw data were not provided in the PMAs. Statistical tests, including the two-way t-test, Chi-square analysis, and Fisher Exact Test, were utilized to determine statistical significance.

## 3. Results

### 3.1. Demographic Comparison

Across all three trials, most subjects identified as Caucasian, non-Hispanic. The age distribution among the control groups did not exhibit any significant differences (*p* = 0.5548): PanOptix 69 ± 6.46, Synergy 68.5 ± 7.7, and Symfony 67.9 ± 7.9. However, there were significant variations in the age distribution among the trial arms: PanOptix 65.8 ± 7.31, Synergy 68.5 ± 7.1, and Symfony 68 ± 7.5. It was found that PanOptix’s patient population was significantly older than that of Synergy and Symfony (*p*-values = 0.003 and 0.014, respectively) with 72.4% of the participants >80 years old. No significant age differences were observed between Synergy and Symfony.

The male-to-female ratio in each of the trial arms did not yield any significant differences (*p* = 0.5025). However, when analyzing the control groups, a significant difference was observed (*p* < 0.0001). The PanOptix control group exhibited a higher male-to-female ratio of 69.3% male participants compared to the other control groups (*p* < 0.001), while no significant difference was found between Synergy with 65.7% female and Symfony with 57% female participants (*p* = 0.1287) (Table 1).

### 3.2. Efficacy

**Figure 1:**
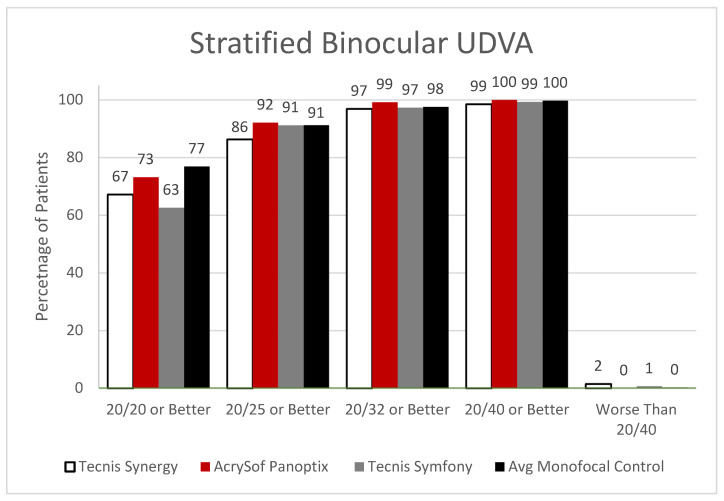
Stratified Binocular Uncorrected Distance Visual Acuity. There was no significant difference between any of the three monofocal/EDOF lenses at any level of acuity. There were no significant differences between the platform’s controls (*p* = 0.055).

Each of the lenses was able to prove non-inferiority to their monofocal control with binocular UDVA, but none of the measurements comparing the trial arms to each other was statistically significant (Figure 1).

**Figure 2:**
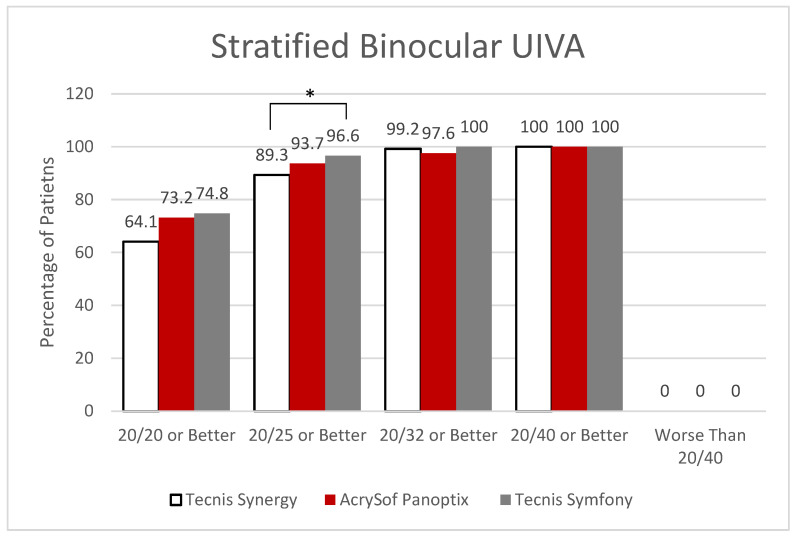
Stratified Binocular Uncorrected Intermediate Visual Acuity. * Statistically Significant.

It was found that 100% of those that received PanOptix could achieve a binocular UDVA of 20/40 or better with 73.2% achieving 20/20 or better compared to Synergy (98.5% at 20/40, 67.2% at 20/20) and Symfony (99.3% at 20/40, 62.6% at 20/20). Symfony demonstrated statistically better outcomes than Synergy (*p* = 0.0182) for patients achieving postoperative visual acuity of 20/25 or better with binocular UIVA (96.6% vs. 89.3%, respectively). However, the relationships between Symfony and PanOptix, as well as Synergy and PanOptix, were insignificant for UIVA (Figure 2).

**Figure 3:**
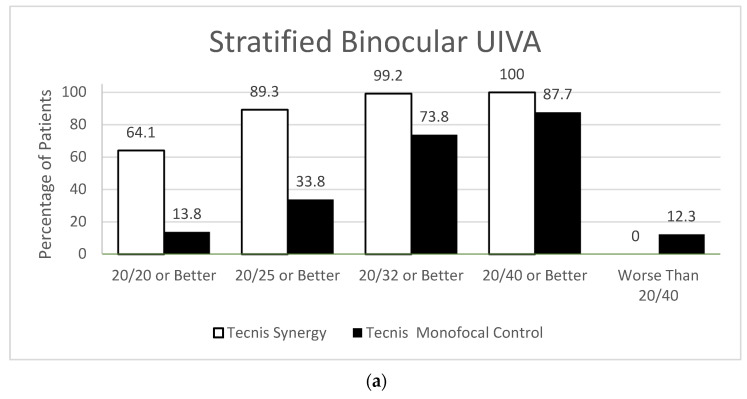

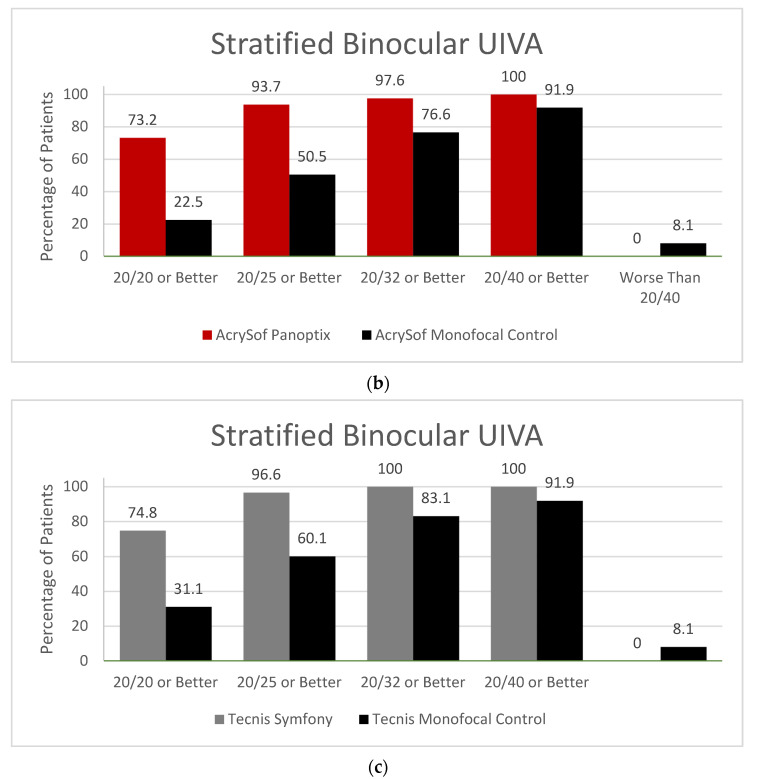
Stratified Binocular Uncorrected Intermediate Visual Acuity Compared to Controls, (**a**) Synergy vs. control, (**b**) PanOptix vs. control, (**c**) Symfony vs. control.

Each of the lenses outperformed their monofocal controls with binocular UIVA. PanOptix showed 73.2% vs. 22.5% of control achieving 20/20 or better compared to 64.1% of Synergy vs. 13.8% of control and 74.8% of Symfony vs. 31.1% of control (Figure 3).

**Figure 4:**
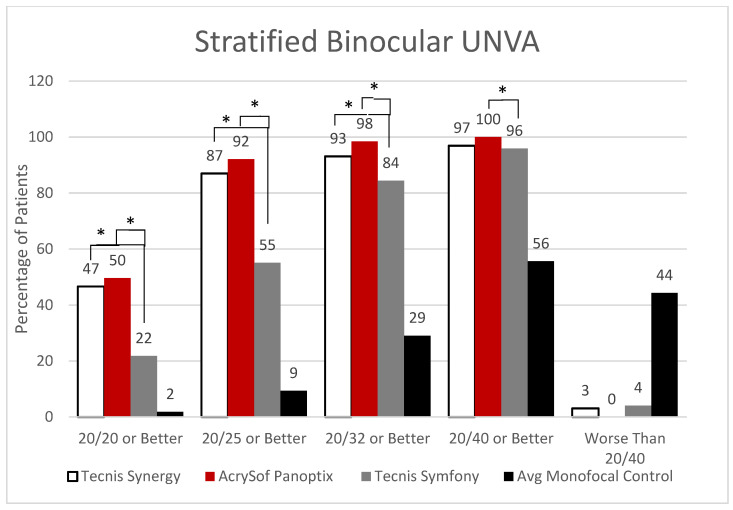
Stratified Binocular Uncorrected Near Visual Acuity IOL Comparison. Synergy vs. Symfony (*p* < 0.0001 for 20/20), (*p* < 0.0001 for 20/25), (*p* = 0.0243 for 20/32) and PanOptix vs. Symfony (*p* < 0.0001 for 20/20), (*p* < 0.0001 for 20/25), (*p* < 0.0001 for 20/32), (*p* = 0.032 for 20/40). No significant differences between the platform’s controls (*p* = 0.212). * Statistically Significant.

All three multifocal/EDOF lens types outperformed their monofocal controls with binocular UNVA, but when compared to each other, both Synergy and PanOptix consistently outperformed Symfony. When compared to 22% of Symfony participants achieving 20/20 or better with UNVA, Synergy had significantly better outcomes at 47% (*p* < 0.0001). This is also reflected with those achieving 20/25 as well as 20/32 or better (*p* < 0.0001, *p* = 0.0243, respectively) but was insignificant at 20/40 or better (*p* = 0.7536). PanOptix achieved significantly better UNVA outcomes at all levels measured when compared to Symfony: 50% vs. 22% at 20/20 or better (*p* < 0.0001), 92% vs. 55% at 20/25 or better (*p* < 0.0001), 98% vs. 84% at 20/32 or better (*p* < 0.0001), and 100% vs. 96% at 20/40 or better (*p* = 0.0320). Average monofocal controls showed 9% of patients achieving a UNVA of 20/25 or better and only 2% at 20/20 or better (Figure 4).

**Figure 5:**
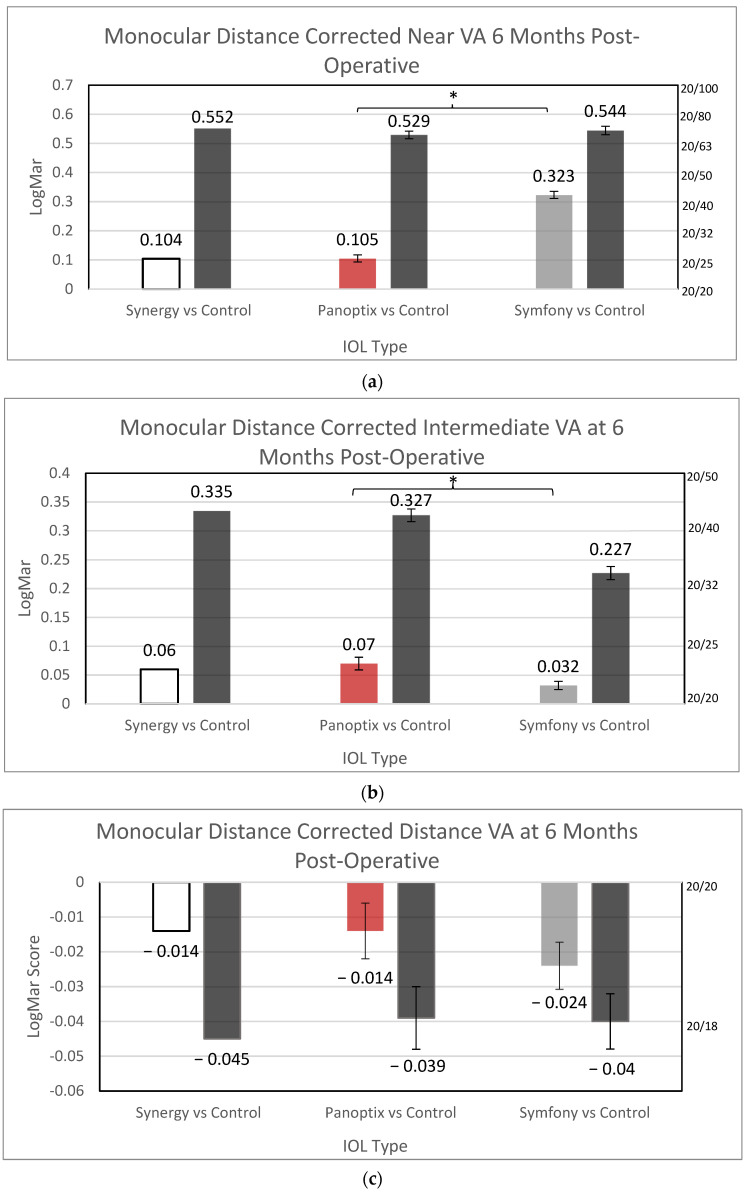
Monocular Distance-Corrected Visual Acuities 6 Months Post-operative, (**a**) Distance corrected near, (**b**) Distance corrected intermediate, (**c**) Distance corrected distance, standard deviations noted if provided by respective PMA data. VA = Visual acuity, * Statistically Significant.

After 6 months post-operative, Symfony with a mean monocular distance CIVA of 0.032 logMAR provided better outcomes than PanOptix with a mean visual acuity of 0.07 logMAR (*p* = 0.0032). PanOptix with a distance CNVA of 0.105 logMAR showed statistically better outcomes than Symfony recorded at 0.323 logMAR (*p* < 0.0001). There was no difference between these two IOLs for CDVA (Figure 5). Synergy could not be compared to the other lenses at any distance because they did not provide a measure of variance in their study regarding these metrics. Additionally, mean monocular and binocular UDVA, UIVA, and UNVA were recorded for Symfony: monocular (0.114 ± 0.142, 0.087, and 0.241 ± 0.142 logMAR, respectively) and binocular (0.034 ± 0.106, 0.002 ± 0.085, and 0.146 ± 0.112 logMAR, respectively). Synergy reported all binocular values: 0.023, 0.22, and 0.06 logMAR, respectively, but only monocular UDVA of 0.09 logMAR. It is important to note that standard deviations were not reported on each mean, and no values were reported for PanOptix.

### 3.3. Defocus Curves

**Figure 6:**
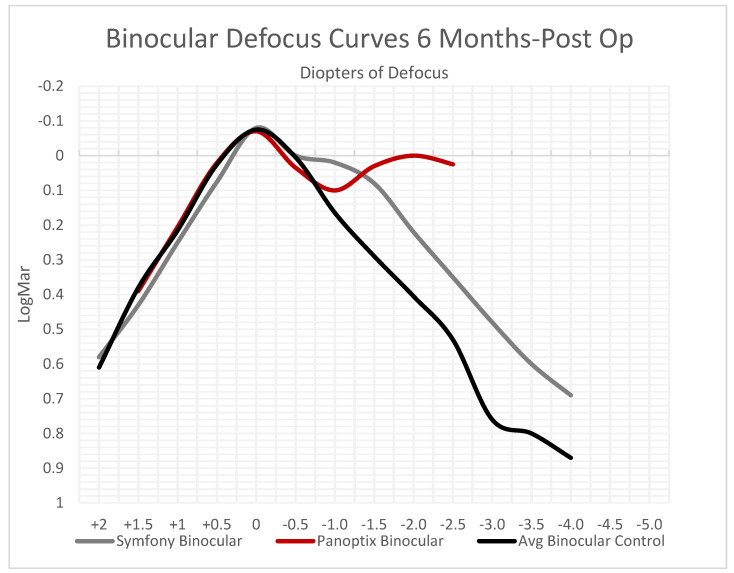
Binocular Defocus Curves 6 Months Postoperatively. Symfony and PanOptix both had higher acuity at larger negative values.

**Figure 7:**
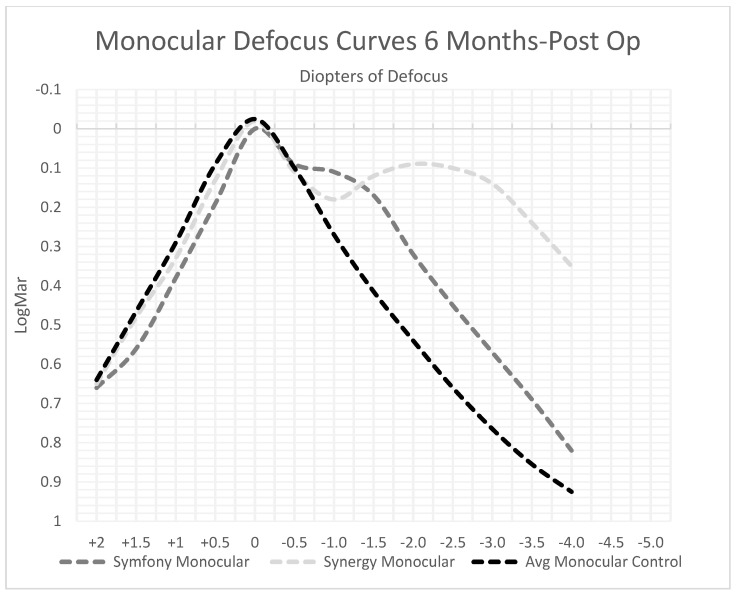
Monocular Defocus Curves 6 Months Postoperatively. Both Symfony and Synergy show that at larger negative values of defocus, their IOLs performed better than the control.

Based on the available data, it was observed that Symfony and PanOptix exhibited better binocular acuity with increased degrees of defocus compared to their monofocal controls (Figure 6). Symfony specifically had >0.5 D of increased focus range (95% CI). A similar trend was observed in monocular acuity for Symfony and Synergy patients (Figure 7). The defocus curves for each lens suggest that multifocal/EDOF lenses function effectively at distance much like an average monofocal control lens but continue to provide satisfactory visual outcomes at intermediate and near distances.

### 3.4. Contrast Sensitivity

Synergy showed a reduction in mesopic contrast sensitivity up to 0.448 log compared to the controls with a reduction of up to 0.392 log. PanOptix was consistently outperformed by its monofocal control, however, this difference was not deemed significant as all means were within 1 line. At 12 cpd, Symfony, compared to its control, showed a median difference of −0.16 log mesopic without glare, −0.170 log mesopic with glare and −0.215 log photopic with glare, failing to demonstrate non-inferiority. While these multifocal/EDOF lenses do not perform as well compared to their respective controls, each PMA notes that these differences in contrast sensitivity are not clinically significant.

### 3.5. Toricity

Toric models of the multifocal/EDOF lenses are available; however, specific outcomes were not measured in the PMAs. Each manufacturer referred to their previous PMAs as evidence of the efficacy and safety of their respective toric lenses. It should be noted that no significant differences were observed in the cylinder values between the experimental arms of the lenses: PanOptix 0.484D ± 0.270, Symfony 0.505D ± 0.248, and Synergy 0.437D ± 0.247 (*p* = 0.5633) (Table 1). None of the multifocal/EDOF lenses demonstrated statistically significant differences in cylinder values compared to their respective controls.

PanOptix conducted a sub-study where four types of astigmatism were induced (1.0 and 1.5D, with and against the rule) in their patients. Visual acuity measurements were then taken monocularly and binocularly under these induced astigmatic conditions. The results indicated that the induced astigmatism caused a maximum LogMAR reduction of 0.28 with UDVA, which was less than one line worse than the monofocal control. With UIVA, the maximum reduction was 0.14 logMAR, and with UNVA, the maximum reduction was 0.12 logMAR. When considering the combined intermediate and near acuity, the mean visual acuity under astigmatic blur conditions was less than 0.23 LogMAR.

### 3.6. Spherical Equivalent

Each of the studies included the average target spherical equivalent for the multifocal/EDOF lens and their respective control (Table 1). Symfony targeted a more negative SEQ of −0.203D ± 0.148 than Synergy at −0.009D ± 0.097 and PanOptix at −0.015D ± 0.104 which was significant (*p* < 0.0001). Synergy compared to PanOptix was not found to be statistically different (*p* = 0.6289). Synergy was also noted to be targeting a different spherical equivalent than its respective control: trial −0.009D ± 0.097, control −0.045D ± 0.114 (*p* = 0.0053).

### 3.7. Safety

One of the primary safety outcomes assessed in each study was the need for secondary surgical interventions (SSI) in the first eye postoperatively (Table 2). When comparing these results to the International Standard Organization’s Safety and Performance Endpoints (ISO SPE) benchmark of 0.80%, each IOL demonstrated either equality or superiority to historical standards. Specifically, PanOptix had an SSI rate of 0.8%, Synergy had a rate of 0.7%, and Symfony showed no occurrences of SSI. Loss of preoperative Snellen or logMAR values due to IOL placement was not noted in the three PMAs.

**Figure 8:**
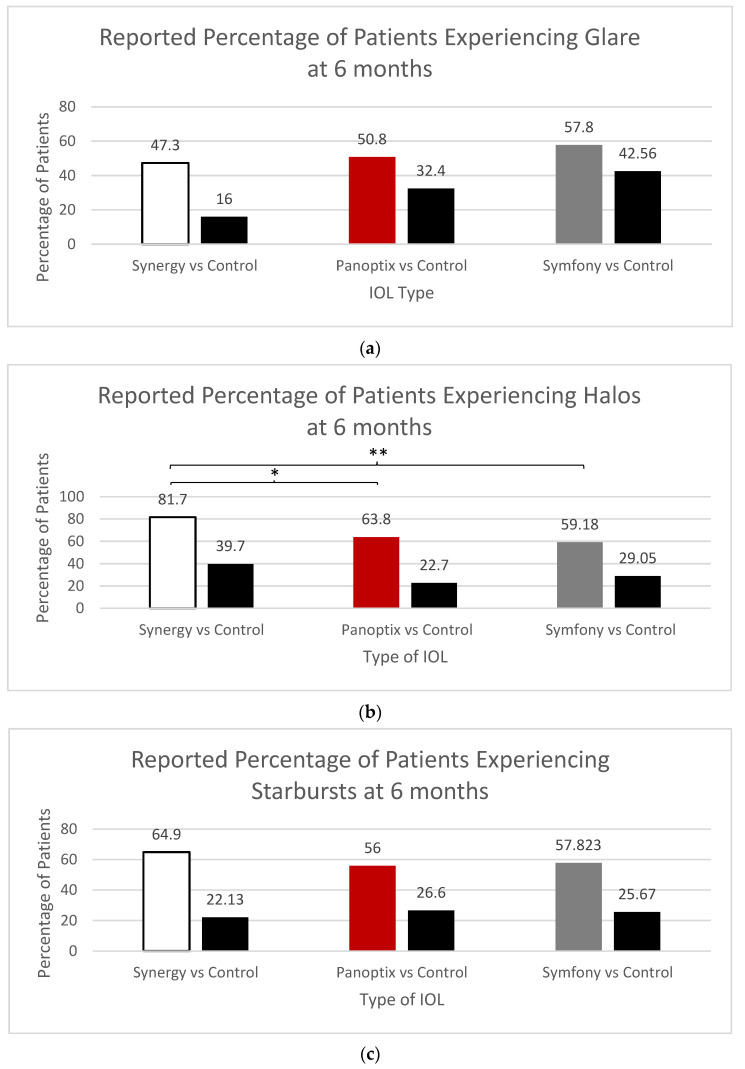
Cumulative Percentage of Patients with Visual Disturbances, (**a**) Reported Percentage of Patients Experiencing Glare, (**b**) Reported Percentage of Patients Experiencing Halos, (**c**) Reported Percentage of Patients Experiencing Starbursts, * and ** Statistically Significant.

Regarding visual disturbances, no significant differences were observed between the three lenses with 50.8% of participants with PanOptix, 47.3% of Synergy, and 57.8% of Symfony experiencing glare. Subjects with the Synergy trifocal lens reported experiencing more halos overall (81.7%) compared to Symfony (63.8%) and to PanOptix (59.18%) (*p* > 0.0001 and 0.0013, respectively). No statistically significant differences were found among the three lenses in relation to starbursts, with reported rates of 56% of PanOptix participants, 64.9% of Synergy, and 57.82% of Symfony (Figure 8).

### 3.8. Patient-Reported Outcomes

**Figure 9:**
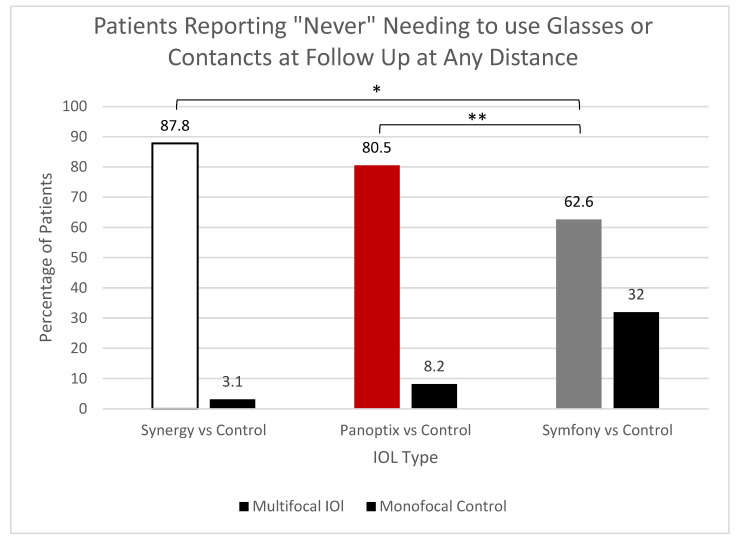
Cumulative Corrective Lens Usage Postoperatively. Symfony vs. Panoptix (*p* = 0.0013) and Symfony vs. Synergy (*p* < 0.0001). Statistical comparisons were not made between each platform and their monofocal control. * and ** Statistically Significant.

Another metric compared among the three lenses was the postoperative need for corrective lenses. The values for those who required corrective lens use and those who did not require glasses postoperatively were aggregated for each trial IOL and compared (Figure 9).

Overall, 87.8% of participants with Synergy and 80.5% of those with PanOptix reported “Never” needing glasses or contact lenses exhibiting significantly lower levels of corrective lens use compared to 62.6% of those with Symfony (Synergy vs. Symfony *p* < 0.0001; PanOptix vs. Symfony *p* = 0.0013). Statistical comparisons were not made between the IOLs and their respective controls.

### 3.9. OptiBlue

Symfony has since developed a new lens, Symfony OptiBlue, that did not have a standalone PMA. Tecnis claims that this InteliLight technology meets the same EDOF goals with better performance in low light and contrast. A clinical study is included below [23]:

In the clinical study of the Tecnis OptiBlue IOL (Model ZV9003), it was found that 100% of the OptiBlue first eyes and 99.2% of second eyes were 20/40 or better for best-corrected distance visual acuity, exceeding the FDA grid rate of 92.5%. At one-year post-operative, 82.3% of first eyes had achieved a best-corrected distance visual acuity of 20/20 or higher, with all eyes achieving a best-corrected visual acuity of 20/40 or better, exceeding the FDA grid rate of 96.7%.

The Ishihara color test and the Farnsworth–Munsell D-15 color vision tests were used to measure binocular color vision. It was found that at one year post-operative, 99.2% of subjects implanted with the OptiBlue IOL passed the color vision test, compared to 95.8% of subjects implanted with the control lens.

Statistically significant differences between the OptiBlue and control groups were found for driving during the day. At 4–6 months post-operative, there were no OptiBlue IOL recipients who reported having difficulty while driving during the day, while 7.1% of the control lens recipients reported a level of difficulty (*p* = 0.0044). At 1 year, 1.9% of OptiBlue IOL recipients reported having a level of difficulty driving during the day, compared to 8.3% of control lens recipients (*p* = 0.0330). Regarding driving at night, mean night driving visibility was lower for OptiBlue than the monofocal control though not noted to be statistically significant. In both rural and urban environments (background interaction and visual clutter), the mean visibility loss was within 25% between the monofocal control and OptiBlue.

There were no persistent adverse events among first eyes reported during the study, and only one (1/126) of the second eyes presented with a secondary surgical intervention, comparing favorably to reported FDA rates of adverse events.

## 4. Discussion

Both PanOptix and Symfony showed superiority to Synergy for near acuity. PanOptix performed as the superior lens regarding the primary measured outcome of monocular distance CNVA at 6 months post-operative. As trifocal IOLs, Synergy, and PanOptix showed similar outcomes across all three tested focal points. Symfony demonstrated the best performance at distance CIVA, which aligns with its design as an EDOF lens optimized for intermediate vision. However, it should be noted that the absence of variance measures prevents the evaluation of statistical significance for these values.

Several previous studies have examined the differences among these lenses and have reported varying results. For instance, studies by Ferreira et al. [4] and Scheepers et al. [8] found no statistical difference between Synergy and PanOptix for near or intermediate monocular distance corrected vision. Similarly, a study by Shin et al. [5] showed that Synergy outperformed Symfony at near and intermediate acuities (*p* < 0.001) 3 months postoperatively. Additionally, studies conducted by Ruiz-Mesa et al. [6] and Farvardin et al. [7] demonstrated that PanOptix performed better than Symfony in terms of intermediate (*p* < 0.001, *p* = 0.089, respectively) and near visual acuity (*p* < 0.001, *p* = 0.001).

Symfony reported the lowest rate of complete independence from glasses and contacts at 62%, although this was still significantly higher than its respective control. In comparison, the two trifocal lenses, PanOptix and Synergy, reported independence values of 80% and 87%, respectively. A smaller study by Blehm et al. [9] reported more than 90% independence from glasses and contacts in their PanOptix group, although the sample size was limited to 30 patients. 

These results suggest that Symfony provides high acuity for UIVA but performs relatively worse for UNVA compared to the other platforms. A study by Miháltz et al. [24] found that some of the factors that seem to be impacting Symfony’s performance with near visual acuity are larger pupils in addition to uncorrected cylindrical error. These findings are consistent with a study by Sudhir et al. [10], which identified two distinct peaks in the defocus curve of Symfony, corresponding to intermediate and distance acuity. Mirroring these findings, Gill et al. [25] found that Symfony seems to be the best choice for intermediate acuities. In contrast, the PanOptix defocus curve did not exhibit these peaks and demonstrated better acuity at both UNVA and UDVA. In the present study, Synergy and PanOptix performed similarly across all three focus points.

Although a direct comparison of contrast sensitivity between the multifocal/EDOF lenses was not possible, it was consistently lower in all the lenses, with PanOptix and Synergy stating that the differences were not clinically significant. Overall, the clinical studies consistently reported an increase in visual disturbances and a reduction in contrast sensitivity when comparing the new IOLs to their respective controls. These findings align with the well-known limitations of multifocal/EDOF IOLs [11,26]. A study by Moshirfar et al. [27] found that night vision disturbances were increased in Synergy at 1 and 3 months post-operative (*p* = 0.01, 0.03) while at 6 months post-operative, patients with PanOptix reported more (*p* = 0.02). However, a study by Doroodgar et al. [28] found that both Symfony and PanOptix performed better (*p* < 0.001) than another multifocal, ReSTOR regarding these visual disturbances.

Visual disturbances varied among the experimental IOLs, halos being most reported with the Synergy trifocal lens (Figure 8b). However, there were no significant differences in the occurrence of glare and starbursts between the three platforms. A study by Dr. Sondra Black [29] followed a small group of 50 patients who had their dominant eye implanted with Symfony and their non-dominant eye implanted with a Tecnis MF (multifocal) low add +3.25 IOL and it was found that at month three, a majority of patients noted no halos, glare or starbursts (96.6%, 100% and 100%, respectively).

Moving forward, it is valuable to promote greater standardization from the FDA when compiling data from companies into PMAs. This could include the requirement for contrast sensitivity and defocus curves to be generated under consistent conditions (e.g., photopic/mesopic, with or without glare, and binocular/monocular). Additionally, there should be uniformity in the range of defocus curves examined. As observed in Figure 6, PanOptix assessed defocus up to −2.5 diopters, while other platforms extended it to −4.0 or −5.0 diopters. Establishing a standardized range would enable far better comparisons.

Standardization of visual acuity measurements is also crucial. All cases should report uncorrected and corrected acuity at near, intermediate, and distance using mean logMAR or Snellen, both binocularly and monocularly. Furthermore, it would be beneficial for companies to report the number of patients achieving 20/20, 20/25, etc., or better vision under the same conditions. Employing a standardized questionnaire to assess visual disturbances and the need for glasses/contacts would allow clinicians to make more informed decisions regarding the most suitable IOLs for their patients. While additional data provided by the platforms would be valuable, we strongly recommend the aforementioned measures.

The newer multifocal/EDOF lenses demonstrated successful achievement of their endpoints, offering improved near and intermediate visual acuity while maintaining distance acuity. However, compared to their monofocal controls, these lenses are associated with a higher incidence of visual disturbances, such as glare, halos, and starbursts. Additionally, there may be a slight decrease in contrast sensitivity [30]. Nonetheless, multifocal/EDOF lenses represent significant advancements in promoting complete independence from glasses and contact lenses compared to their monofocal counterparts. When comparing the different multifocal/EDOF lenses to each other, Synergy and PanOptix exhibit similar performance, providing high acuity at all three focus points. On the other hand, Symfony shows lower efficacy at UNVA but excels with UIVA. Symfony is the oldest lens among the three, and the newer OptiBlue lens shows significant improvement and superiority over its monofocal control.

Our primary objective is to assist surgeons in promoting transparency when educating their patients about the available IOL options. We firmly believe that patients should have access to quantitative data when making informed decisions about their lens options. The results compiled in this article can be utilized to educate patients about the statistical outcomes observed for UDVA, UIVA, and UNVA in relation to each lens. Patients can note that these lenses provided 20/20 vision for 63–73% of participants for UDVA, 64–75% for UIVA, and 22–50% for UNVA depending on the specific lens. Additional data can also be provided to reassure patients about the safety of these lenses. By presenting patients with statistically significant results on independence from corrective lenses and potential visual disturbances, patients can make choices based on comparative data that supports their specific lifestyles and preferences.

### Limitations

In terms of the limitations of this study, some comparisons were not made due to a lack of common metrics across the three platforms. While part of this is due to the companies’ different goals for their respective lenses, it was also in part from a non-universality in the PMA process. While each of the PMA’s focused on the same general themes such as safety and efficacy, the specific metrics assessed were not standardized.

For instance, Synergy did not provide any measure of variance when reporting their mean logMAR values. PanOptix did not present their average logMAR scores for UDVA, instead grouping the data based on the percentage of individuals achieving a certain level of Snellen vision (e.g., xx/20 or better). On the other hand, Symfony had the most comprehensive data among the three IOLs examined. These discrepancies in reporting methods, although not problematic within each study, pose challenges for clinicians trying to make informed decisions about the best lens for their patients. Standardizing the required data presentation would greatly assist in this regard.

Additionally, these PMAs do not provide long-term data for their participants. Clinicians and patients could benefit from gathering 1+ years of post-operative data for UDVA, UIVA, and UNVA, giving them an even wider scope when choosing an appropriate lens.

## 5. Conclusions

Promoting transparency in educating patients about available multifocal and EDOF IOL options, while facilitating data-supported informed decision-making are key goals of this article. Additionally, we affirm that standardized methods of data gathering and reporting for PMAs are crucial for an even more comprehensive analysis.

## Figures and Tables

**Table 1:** Demographic and Pre-Operative Data Comparison.

	PanOptix Trial	PanOptix Control	Synergy Trial	Synergy Control	Symfony Trial	Symfony Control
Subject Number	*n* = 129	*n* = 114	*n* = 135	*n* = 137	*n* = 148	*n* = 151
Age Mean ± SD	65.8 ± 7.31	69 ± 6.46	68.5 ± 7.1	68.5 ± 7.7	68 ± 7.5	67.9 ± 7.9
Range	(44, 81)	(48, 86)	(48, 85)	(46, 92)	(46, 86)	(47, 88)
Age groups: <60	Reported as <65	Reported as <65	17 (12.6%)	17 (12.4%)	15 (10.1%)	24 (15.9%)
60–69	45 (34.9%)	22 (19.3%)	50 (37%)	57 (41.6%)	70 (47.3%)	61 (40.4%)
70–79	Reported as ≥65	Reported as ≥65	62 (45.9%)	56 (40.9%)	52 (35.1%)	56 (37.1%)
>80	84 (65.1%) *	92 (80.7%) *	6 (4.4%)	7 (5.1%)	11 (7.4%)	10 (6.6%)
Male: Female Ratio	44:85	79:35 *	41:94	47:90	57:90	65:86
Pre-operative Keratometric Cylinder	0.484D ± 0.270	0.544D ± 0.267	0.437D ± 0.247	−0.009D ± 0.097	0.505D ± 0.248	−0.203D ± 0.148
Target SEQ	−0.015D ± 0.104	−0.020D ± 0.174	−0.009D ± 0.097	−0.045D ± 0.114 *	−0.203D ± 0.148 *	−0.192D ± 0.152
Iris Color: Blue/gray	NR	NR	42 (31.1%)	37 (27%)	47 (31.8%)	47 (31.1%)
Iris Color: Brown/black	NR	NR	54 (40%)	63 (46%)	63 (42.6%)	63 (41.7%)
Iris Color: Green/hazel	NR	NR	39 (28.9%)	37 (27%)	38 (25.7%)	41 (27.2%)

* Statistically significant, SD = Standard deviation, NR = Not reported, D = Diopters, SEQ = Spherical equivalent, EDOF = Extended depth of focus.

**Table 2:** Serious Adverse Events Compared to ISO SPE.

	6 Month Cumulative Serious Adverse Events First Eye			
	Panoptix (*n* = 129)	Synergy (*n* = 135)	Symfony (*n* = 148)	ISO SPE %
Cystoid Macular Edema	0	1 (0.7%)	1 (0.7%)	3%
Secondary Surgical Intervention	1 (0.8%)	1 (0.7%)	0	0.80%
Retinal Tear	1 (0.8%)	0	0	NR
Pupillary Block	0	0	0	0.10%
	**6 Month Cumulative Serious Adverse Events Second Eye**			
	**Panoptix (*n* = 127)**	**Synergy (*n* = 135)**	**Symfony (*n* = 148)**	**ISO SPE %**
Cystoid Macular Edema	1 (0.8%)	1 (0.7%)	1 (0.7%)	3%
Secondary Surgical Intervention	2 (1.6%)	3 (2.2%) *	2 (1.4%) *	0.80%
Retinal Tear	0	0	0	NR
Retinal Detachment	0	0	0	0.30%
Pupillary Block	0	0	0	0.10%
Device Dislocation	1 (0.8%)	0	0	NR
Vitreous Prolapse	1 (0.8%)	0	0	NR
Hypopyon	0	1 (0.7%)	1 (0.7%)	0.30%
Endopthalmitis	0	0	1 (0.7%)	0.10%

* Not all associated with device properties, NR = Not Reported.

## Data Availability

All data analyzed during this study are included in this published article as citations in the results section.
